# Effects of lipid‐based nutrient supplements or multiple micronutrient supplements compared with iron and folic acid supplements during pregnancy on maternal haemoglobin and iron status

**DOI:** 10.1111/mcn.12640

**Published:** 2018-07-26

**Authors:** Josh M. Jorgensen, Per Ashorn, Ulla Ashorn, Lacey M. Baldiviez, Austrida Gondwe, Ken Maleta, Minyanga Nkhoma, Kathryn G. Dewey

**Affiliations:** ^1^ Program in International and Community Nutrition, Department of Nutrition University of California Davis California USA; ^2^ Department of Paediatrics Tampere University Hospital Tampere Finland; ^3^ Faculty of Medicine and Life Sciences University of Tampere Tampere Finland; ^4^ Center for Child Health Research University of Tampere School of Medicine and Tampere University Hospital Tampere Finland; ^5^ Department of Community Health University of Malawi College of Medicine Blantyre Malawi

**Keywords:** haemoglobin, iron, lipid‐based nutrient supplement, multiple micronutrient, pregnancy

## Abstract

We examined the effect of three types of prenatal supplements containing different amounts of iron on haemoglobin (Hb) and iron status (zinc protoporphyrin [ZPP] and soluble transferrin receptor [sTfR]) in late pregnancy among 1,379 women in rural Malawi. Participants were recruited at ≤20 gestational weeks (gw) and randomly assigned to consume daily (1) 60‐mg iron and folic acid (IFA); (2) 20‐mg iron plus 17 micronutrients in a capsule (MMN); or (3) lipid‐based nutrient supplement (LNS; 118 kcal) with 20‐mg iron plus 21 micronutrients, protein, and fat. We analysed differences between intervention groups in mean Hb, ZPP, and sTfR at 36 gw, and the proportion with anaemia (Hb < 100 g L^−1^) and iron deficiency (ZPP > 60 μmol mol^−1^ haem or sTfR > 6 mg L^−1^) at 36 gw. Women in the IFA group had higher Hb at 36 gw than women in the LNS group (*P* = 0.030) and higher iron status (lower ZPP and sTfR) than women in both the LNS (*P* < 0.001 for both ZPP and sTfR) and MMN (*P* = 0.025 and *P* = 0.046) groups. Results for anaemia and iron deficiency showed similar trends. Further research is needed to elucidate the appropriate amount of iron to improve Hb and iron status, while improving birth outcomes.

**Key messages**

- Pregnant Malawian women who consumed 60‐mg iron per day in an iron–folic acid supplement from ≤20 gestational weeks had higher Hb and markers of iron status at 36 gestational weeks than did women who consumed 20 mg day^−1^ as a lipid‐based nutrient supplement or a multiple micronutrient capsule.
- There were no differences in prevalence of anaemia or iron deficiency anaemia between the three groups at 36 gestational weeks.
- Further research is needed to elucidate the optimal dose of supplemental iron during pregnancy in this population.

**Abbreviations**
AGP: α‐1‐acid glycoprotein
CRP: C‐reactive protein
Hb: haemoglobin
IFA: iron–folic acid
LNS: lipid‐based nutrient supplement
MMN: multiple micronutrient
sTfR: soluble transferrin receptor
ZPP: zinc protoporphyrin

## 1 INTRODUCTION

Anaemia during pregnancy is a risk factor for preterm birth and low birthweight, in addition to maternal and infant death (Allen, 2000; New & Wirth, 2015). The global prevalence of anaemia among pregnant women is estimated to be 19.2% (World Health Organization [WHO], 2015), whereas in sub‐Saharan Africa, an estimated 57% of pregnant women are anaemic (Soares Magalhaes & Clements, 2011). Although there are multiple etiologies for anaemia, including micronutrient deficiencies, haemoglobinopathies, and acute and chronic infections, one of the most prevalent causes of anaemia is iron deficiency (Crawley, 2004).

Iron supplementation during pregnancy is helpful in preventing iron deficiency. A recent Cochrane review reported a 70% reduction in maternal anaemia and a 57% reduction in iron deficiency at term among women who received preventive iron supplementation during pregnancy (Pena‐Rosas, De‐Regil, Garcia‐Casal, & Dowswell, 2015). However, the optimal dose of iron is still not known. The WHO recommends 30–60 mg of elemental iron per day, with a preferred daily dose of 60 mg day^−1^ in areas where anaemia among pregnant women is a severe public health problem (WHO, 2012). The UNICEF/WHO/UNU international multiple micronutrient preparation for pregnant and lactating women provides the Recommended Dietary Allowance (RDA) of 15 vitamins and minerals, including 30 mg of iron. A daily supplement with 30 mg (rather than 60 mg) of iron was chosen for multiple reasons (lower side effects with a daily dose of 30 vs. 60 mg; other vitamins in the supplement enhance iron absorption; the need for higher doses of zinc with higher doses of iron would further exacerbate side effects; and the ability to use higher doses of iron in conjunction with the supplement for cases of more severe anaemia; Adu‐Afarwuah et al., 2016; UNICEF/UNU/WHO, 1999). However, even supplementation with only 30 mg of iron has been associated with side effects (Pena‐Rosas, De‐Regil, Dowswell, & Viteri, 2012).

The International Lipid‐based Nutrient Supplement (iLiNS) Project (http://www.ilins.org/) was designed to study the impact on maternal and infant health of supplementation with a small‐quantity lipid‐based nutrient supplement (SQ‐LNS) provided to pregnant and lactating women and their children from 6 to 18 months of age. LNS differ from micronutrient supplements or fortificants because they are food products and contain energy, protein, and essential fatty acids, as well as a wider range of micronutrients than most micronutrient supplements or fortificants, including several macrominerals required for growth. The iron content per daily dose of 20 g of the SQ‐LNS used in this study was set at 20 mg, as described previously (Arimond et al., 2015), which is lower than the 30 mg in the UNICEF/WHO/UNU international multiple micronutrient preparation supplement. Supplementation with 20 mg day^−1^ had been shown to be adequate to prevent iron deficiency anaemia during pregnancy (even among women who were anaemic at entry into prenatal care), while not causing gastrointestinal upset commonly associated with higher doses of iron (Milman, Byg, Bergholt, Eriksen, & Hvas, 2006; Zhou, Gibson, Crowther, & Makrides, 2009). The RDA for iron during pregnancy is 27 mg day^−1^, which drops to 9 mg day^−1^ during lactation (Food and Nutrition Board Institute of Medicine, 2002). Pregnant women in the Mangochi District in Malawi consume an estimated 16–18 mg of iron per day (Hjertholm et al., 2018; Ndekha, 1998). Although this is higher than the median intake among pregnant women in the United States (approximately 15 mg day^−1^; Food and Nutrition Board Institute of Medicine, 2002), it is still well below the RDA. We estimated that consuming a supplement with 20 mg day^−1^, combined with iron from the diet, would provide sufficient iron during pregnancy (without greatly exceeding the RDA during lactation), based on data indicating that iron absorption in the third trimester of pregnancy is high—25–66% when a modest dose of iron (6–18 mg) is consumed daily with food (Barrett, Whittaker, Williams, & Lind, 1994; Whittaker, Barrett, & Lind, 2001). Women in the second trimester need approximately 4–5 mg day^−1^ of absorbed iron, which increases to 5–6 mg day^−1^ in the third trimester (Food and Nutrition Board Institute of Medicine, 2002). Assuming at least 10% absorption of iron from food (the estimated bioavailability from vegetarian diets; Food and Nutrition Board Institute of Medicine, 2002), we calculated that at least 1.5‐mg iron per day would be absorbed from the diet. Assuming 25% of the iron in LNS is absorbed, 20 mg of iron in LNS would provide at least 5 mg day^−1^ of absorbed iron, which together with at least 1.5 mg of dietary iron would be sufficient to meet the needs in late pregnancy when demand is highest.

The primary aim of the iLiNS Project was to examine the effect of SQ‐LNS on birth outcomes and infant growth. Additionally, we have examined a wide range of secondary outcomes among pregnant women and infants, including haemoglobin (Hb) and iron status among pregnant Ghanaian women (Adu‐Afarwuah et al., 2016). In the present study, we examine the effect of three iron‐containing supplements (iron and folic acid [IFA], multiple micronutrient [MMN] capsule, and SQ‐LNS) on Hb and markers of iron status (zinc protoporphyrin [ZPP] and soluble transferrin receptor [sTfR]) among pregnant Malawian women.

## 2 METHODS

The iLiNS Project DYAD Malawi trial was a randomized, controlled, outcome assessor‐blinded supplementation trial of mother–child dyads in the Mangochi District of rural Malawi (iLiNS‐DYAD‐M). The study has been described in detail elsewhere (Ashorn et al., 2015). The primary study focused on the effect of intervention on newborn outcomes and child growth. We have published or plan to publish various articles on other nutrient biomarkers and other outcomes. The focus of the current study is iron deficiency and anaemia. Briefly, study nurses explained the study to women who came to one of the study clinics for antenatal care and who were greater than 15 years of age and no more than 20 gestational weeks (gw). Interested women signed or thumbprinted an informed consent and were enrolled in the study if eligible. Women who had chronic medical conditions, pregnancy complications at enrolment (moderate to severe oedema, blood Hb concentration <50 g L^−1^, systolic blood pressure >160 mmHg, or diastolic blood pressure >100 mmHg), previous enrolment in iLiNS‐DYAD, or concurrent enrolment in another clinical trial were excluded. We enrolled 1,391 women.

Women were randomly assigned to one of three intervention groups in blocks of nine by selecting an opaque envelope that contained one of nine letters. Each intervention group had three letters that corresponded to it. Women in the IFA group were instructed to consume each day from enrolment until delivery a capsule that contained 60 mg of iron and 400 μg of folic acid and from delivery to 6 months post‐partum a placebo capsule. IFA during pregnancy (but not post‐partum) is considered the standard of care in Malawi. Women in the MMN group were instructed to consume each day from enrolment to 6 months post‐partum a capsule that contained 20 mg of iron, in addition to folic acid and 16 additional micronutrients (Table S1). The IFA/placebo and MMN capsules were identical in appearance. Women in the SQ‐LNS group were instructed to consume each day from enrolment to 6 months post‐partum a 20‐g dose of LNS that contained the same 18 micronutrients as the MMN capsule, as well as four additional minerals, protein, and fat, and also provided 118 kcal of energy. Fifteen supplement doses were delivered by study staff every 14 days. Women in the IFA and MMN groups were instructed to consume the capsules with water after a meal, whereas those in the LNS group were instructed to mix one sachet of LNS with a small amount of food consumed as one dose in the morning. The LNS used in this study was deemed acceptable in the study catchment area (Phuka et al., 2011). On the same days that supplements were delivered, any remaining supplements from the previous delivery were counted and collected. The capsules were manufactured by DSM Nutritional Products South Africa (Pty) Ltd (Isando, South Africa). The LNS was produced and packed by Nutriset S.A.S. (Malaunay, France). The capsules and LNS were stored in a dark environment at 20–40°C. Field workers who delivered supplements were the only study staff who knew which women received LNS (but they did not know the difference between MMN and IFA), and participants were instructed not to disclose information about their supplements to anyone other than the field workers. Besides field workers who delivered supplements, all study staff, laboratory staff, and statisticians were blinded to group allocation. A statistician not involved in iLiNS‐DYAD‐M maintained the intervention code, which was stored sealed and not broken until all laboratory and statistical analyses were performed.

At the enrolment visit, sociodemographic information was collected by trained study staff. Trained anthropometrists measured the participants' weight and height in triplicate using high‐quality scales (SECA 874 flat scale, Seca GmbH & Co., Hamburg, Germany) and stadiometers (Harpenden stadiometer, Holtain Limited, Crosswell, Crymych, UK). Peripheral malaria parasitaemia was measured with a rapid test kit (Clearview Malarial Combo, Alere, South Africa), and HIV was analysed using a whole‐blood antibody rapid test (Alere Determine HIV‐1/2, Alere, South Africa) and using another whole‐blood antibody rapid test (Uni‐Gold HIV, Trinity Biotech plc, Bray, Ireland).

At the enrolment and 36 gw planned study visits, clinic nurses collected blood from the antecubital vein into a 7.5‐ml trace mineral‐free polypropylene syringe (Sarstedt Monovette, NH4‐heparin, Sarstedt Inc., Newton, NC, USA). The blood tube was immediately inverted 10 times to mix the heparin anticoagulant with the blood to prevent clotting. A small aliquot of the whole blood was pipetted out and used to analyse Hb on the Hemocue 201+ system (Hemocue, Brea, CA, USA). The tube containing the remaining whole blood was then placed in an insulated cooler with ice packs until processing. Trained lab staff then aliquoted whole blood into microcuvettes and washed the red cells 3 times. The washed red cells were used for ZPP analysis (Aviv hematofluorometer, Aviv Biomedical Inc., Lakewood, NJ, USA). Trained lab staff then centrifuged the whole blood at 3,000 RPM for 15 min and separated plasma into storage cryovials. The storage vials were placed upright in freezer boxes in a −20°C freezer for temporary storage at the satellite clinics. Within 48 hr, drivers transported the plasma to the main laboratory for long‐term storage at −80°C.

Plasma was shipped to UCD on dry ice (World Courier) for analysis. We analysed sTfR from those samples by immunoturbidimetry on the Cobas Integra 400 system autoanalyser (F. Hoffmann‐La Roche Ltd., Basel, Switzerland). We analysed all the samples in singlet, except for 5%, which we randomly selected to be analysed in duplicate. None of those samples had a coefficient of variation greater than 5%.

ZPP and sTfR are commonly used markers of iron status. There is an inverse correlation between iron status and ZPP, as during iron deficiency, zinc, instead of iron, is incorporated into protoporphyrin IX, resulting in the formation of ZPP (Braun, 1999). The concentration of sTfR is also inversely proportional to total body iron status, as cells upregulate TfR when iron is needed in cells. Soluble TfR is proportional to the concentration of cellular TfR (Beguin, 2003). Ferritin is a commonly used marker of iron status. However, we did not analyse serum or plasma ferritin because we expected a high prevalence of inflammation in the study population, which complicates the interpretation of ferritin measurements.

Anaemia was defined as Hb <100 g L^−1^, which has been suggested as an appropriate cut‐off for pregnant women of African descent (Cao & O'Brien, 2013; Chang, O'Brien, Nathanson, Mancini, & Witter, 2003; Johnson‐Spear & Yip, 1994). In exploratory analyses, we also examined differences between groups in proportion of women with anaemia and iron deficiency anemia (IDA) using an Hb cut‐off of 110 g L^−1^ (WHO, 2011). High Hb was defined as >130 g L^−1^ (Pena‐Rosas et al., 2015). Iron deficiency was defined as ZPP >60 μmol mol^−1^ haem (Walsh et al., 2011) or sTfR >6.0 mg L^−1^. An sTfR cut‐off of 8.5 mg L^−1^ has been used previously when analysing sTfR by the enzyme‐linked immunosorbent assay (ELISA) method (Carriaga, Skikne, Finley, Cutler, & Cook, 1991; Rusia et al., 1999; Vandevijvere, Amsalkhir, Van Oyen, Egli, & Moreno‐Reyes, 2013). However, Pfeiffer et al. (2007) compared the ELISA and autoanalyser methods and found that the autoanalyser gives sTfR estimates approximately 30% lower than the ELISA method. Therefore, we decreased the 8.5 mg L^−1^ cut‐off by approximately 30%, to 6.0 mg L^−1^. IDA was defined as Hb <100 g L^−1^ and either ZPP >60 μmol mol^−1^ haem or sTfR >6.0 mg L^−1^.

The trial was performed according to Good Clinical Practice guidelines and the ethical standards of the Helsinki Declaration. The protocol was approved by the College of Medicine Research and Ethics Committee, University of Malawi, Institutional Review Board at UC Davis, and the Ethics Committee of Pirkanmaa Hospital District, Finland. Key details of the protocol were published at the clinical trial registry of the National Library of Medicine, Bethesda, MD, USA (http://www.clinicaltrials.gov/, trial identification NCT01239693).

### 2.1 Details of statistical analysis

Details of the sample size calculation are described in detail elsewhere (Ashorn et al., 2015). Briefly, assuming an effect size of 0.3 (difference between groups, divided by the pooled *SD*) for each continuous outcome, assuming 80% power and a two‐sided Type I error rate of 5% would require 216 participants per group, for a total of 648 participants. Allowing for up to 25% loss to follow‐up, we would have needed to recruit 864 subjects. A secondary aim of the primary study was to study the interaction between the maternal intervention and several potential effect modifiers, which required that we increase the sample size. The final sample size of 370 per group provided the study with 80% power to detect main effects of >0.23 *SD*.

We performed statistical analysis with the SAS version 9.3 software package (SAS Institute Inc., Cary, NC, USA). We conducted the statistical analysis according to the analysis plan written and published before the intervention code was opened (http://www.ilins.org). We based the analysis on the principle of modified intention to treat. That is, we included in the analyses all participants who were randomized, except that those with missing data on an outcome variable were excluded from the analyses of that outcome. Two participants whose group allocation was incorrectly transcribed and assigned during enrolment were included in the group corresponding to the actual intervention they received throughout the trial. Outcome variables were inspected for conformance to normal distribution and were transformed where necessary. Soluble TfR, ZPP, C‐reactive protein (CRP), and α‐1‐acid glycoprotein (AGP) were log‐transformed before analyses were performed.

We analysed differences between those included and excluded from the current analysis by Student's *t* test (comparison of means) or Fisher's exact test (comparison of proportions). We analysed the differences between groups in mean Hb, ZPP, and sTfR at 36 gw, with the main effect being intervention group with and without controlling for baseline status of each variable and chosen covariates. Unadjusted analyses were performed using analysis of variance models, whereas analysis of covariance (ANCOVA) was used for unadjusted models. We then performed pairwise comparisons with a Tukey–Kramer adjustment. We compared the differences between groups in the proportion of women who had low or high Hb, high ZPP or sTfR, or who had IDA (i.e., low Hb and either high ZPP or sTfR) at either the baseline or the 36 gw visit by log‐Poisson regression. We examined values at 36 gw with and without controlling for the baseline status for each variable and chosen covariates.

The covariates in the ANCOVA and log‐Poisson regression models were included based on whether these variables (a) have been shown in prior work to influence the outcome and (b) were associated (*P* < 0.10) with the outcome in bivariate analyses. The following baseline variables were selected a priori and were examined as potential covariates: maternal body mass index (BMI) at enrolment, malaria status, HIV status, primiparity, maternal educational achievement, site at enrolment, season of enrolment, maternal Hb (for ZPP and sTfR analyses), and ZPP and sTfR (for Hb analyses).

To examine effect modification, variables were selected a priori based on their expected associations with Hb, ZPP, and sTfR. Two‐way interactions between group assignment and Hb, ZPP, sTfR, CRP, AGP, and BMI at enrolment; maternal educational achievement, HIV status, malaria status at enrolment, and season of and site at enrolment were included separately in the ANCOVA models (for continuous outcomes) or logistic regression (for bivariate outcomes) for Hb, ZPP, and sTfR at 36 gw. Significant interactions (*P* < 0.05) were further examined by stratifying participants into high or low categories for continuous effect modifiers or presence or absence of a predictor for bivariate effect modifiers, in order to understand the nature of the effect modification.

Because of the known effect of inflammation on Hb (Weiss & Goodnough, 2005), we conducted a sensitivity analysis to compare the groupwise differences in mean Hb at 36 gw (by ANCOVA) and proportion of women who were anaemic at 36 gw (by log‐Poisson regression) after excluding cases with elevated CRP or AGP at 36 gw. Both models controlled for baseline Hb, CRP, and AGP. Elevated CRP was defined as CRP >5 g L^−1^, and elevated AGP was defined as AGP >1 mg L^−1^ (Thurnham et al., 2010).

## 3 RESULTS

**Figure 1:**
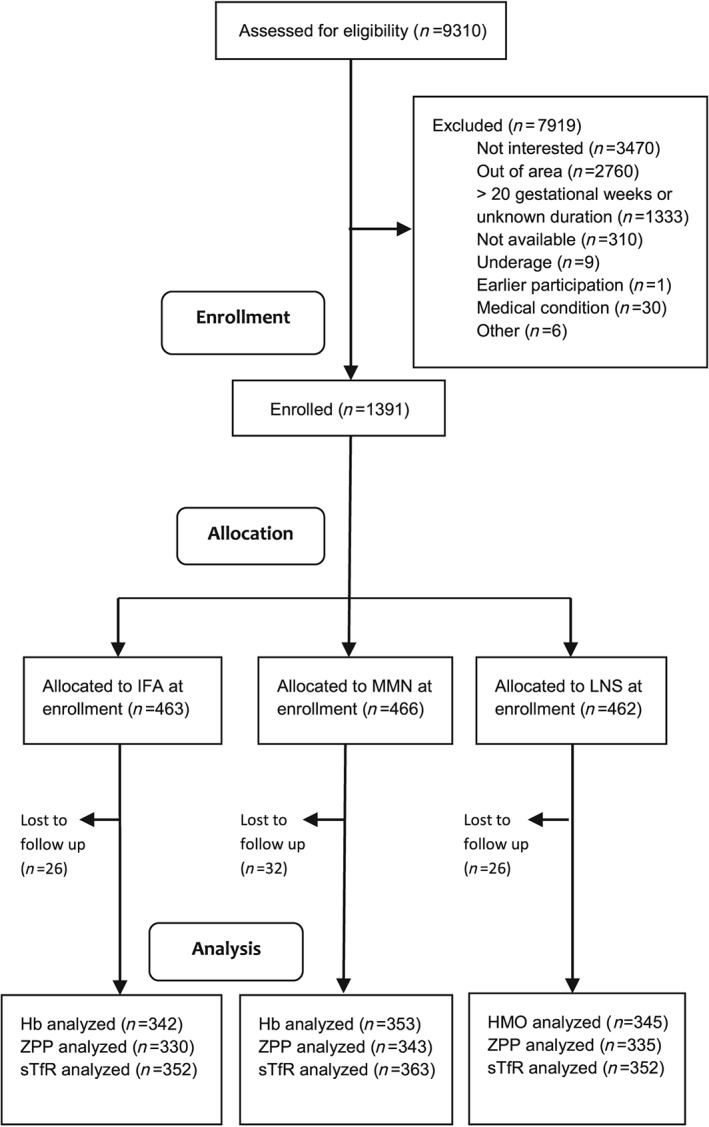
Schematic representation of recruitment, enrolment, and follow‐up of Malawian women who participated in the iLiNS Project. IFA: iron–folic acid; MMN: multiple micronutrient; Hb: haemoglobin; ZPP: zinc protoporphyrin; sTfR: soluble transferrin receptor

Women were enrolled at the four antenatal clinics between February 2011 and August 2012. Of the 9,310 women approached by iLiNS Malawi team members, 1,391 were successfully enrolled and randomized to one of the three intervention groups, with a mean gestational age at enrolment of 16.8 weeks (Figure 1). Twelve of those enrolled carried twins and were excluded from the analyses. Of the remaining 1,379 included in the analyses, we analysed Hb at enrolment from 1,377 (99.9%), ZPP from 1,325 (96.1%), and sTfR from 1,371 (99.4%). At the 36 gw visit, we analysed Hb from 1,040 (75.4% of the original 1,379 women who completed the enrolment visit), ZPP from 1,008 (73.1%), and sTfR from 1,067 (77.4%). There were 352, 363, and 352 participants in the IFA, MMN, and LNS groups, respectively, who were included in the analyses at 36 gw. There were no differences between intervention groups in the proportion of women from whom Hb, ZPP, and sTfR at 36 gw were not available (*P* > 0.8 for all). There were no differences between groups in the mean (*SD*) percentage of days supplements were consumed (IFA, 84.2 [16.6]; MMN, 83.4 [18.1]; LNS, 85.6 [16.9]; *P* = 0.170), severe adverse events (IFA, 9.1%; MMN, 9.7%; LNS, 11.9%; *P* = 0.353), or preterm delivery (IFA, 11.3%; MMN, 9.5%; LNS, 9.1%; *P* = 0.528).

The baseline characteristics of the participants included in the current analyses at 36 gw are shown in Table 1. Compared with those included in the current analyses at 36 gw, those not included in the current analyses were on average younger and of higher socio‐economic status (*P* < 0.001 for both) and had a higher proportion of primiparity and anaemia (*P* < 0.001 for both; Table S2).

**Table 1:** Baseline characteristics of pregnant Malawian women included in analyses at 36 gestational weeks, by intervention group

Characteristic	IFA	MMN	LNS
Number of participants	352	363	352
Mean (*SD*) age (years)	25.1 (5.9)	25.4 (6.1)	25.3 (6.3)
Mean (*SD*) gestational age at enrolment (weeks)	16.8 (2.1)	16.8 (2.1)	16.9 (2.2)
Mean (*SD*) education (completed years)	4.0 (3.4)	4.0 (3.4)	4.1 (3.5)
Mean (*SD*) socio‐economic score	−0.06 (1.0)	−0.04 (0.9)	−0.02 (1.0)
Proportion of nulliparous women	19.4%	19.1%	20.7%
Mean (*SD*) body mass index (BMI; kg m^−2^)	22.1 (2.6)	22.2 (3.0)	22.1 (2.8)
Proportion of women with a low BMI (<18.5 kg m^−2^)	5.4%	5.0%	6.6%
Proportion of anaemic women (Hb <100 g L^−1^)	18.2%	18.0%	19.6%
Proportion of anaemic women (Hb <110 g L^−1^)	45.0%	43.8%	43.4%
Proportion of women with a positive HIV test	14.7%	10.3%	13.1%
Proportion with a positive malaria test (RDT)	21.3%	24.0%	23.1%

*Note*. Hb: haemoglobin; IFA: iron–folic acid; MMN: multiple micronutrients; LNS: lipid‐based nutrient supplement.

At enrolment, the mean (*SD*) Hb level of all participants included in these analyses was 111.5 (16.3) g L^−1^. The prevalence of anaemia (Hb <100 g L^−1^) was 20.8%, whereas 11.1% had high Hb (>130 g L^−1^). The mean (*SD*) ZPP at enrolment was 54.5 (41.6) μmol mol^−1^ haem, with 24.5% of participants having high ZPP (>60 μmol mol^−1^ haem). The mean (*SD*) sTfR was 4.8 (2.7) mg L^−1^, with 19.7% having high sTfR (>6 mg L^−1^).

At 36 gw, the mean (*SD*) Hb was 110.8 (15.2) g L^−1^, 20.4% of women were anaemic, and 8.5% had high Hb. The mean (*SD*) ZPP at 36 gw was 60.2 (40.9) μmol mol^−1^ haem, with 34.0% of participants having high ZPP. The mean (*SD*) sTfR was 5.6 (3.0) mg L^−1^, with 33.5% of participants having high sTfR.

Table 2 shows that there were differences in mean Hb, ZPP, and sTfR between the intervention groups at 36 gw. Results were generally similar whether or not they were adjusted for the baseline value of the outcome variable, and further adjustment for other covariates did not change the findings. After adjusting for baseline Hb, the mean Hb at 36 gw in the IFA group was significantly greater than in the LNS group (*P* = 0.030) and tended to be greater than in the MMN group (*P* = 0.058). Adjusting for baseline ZPP, mean ZPP at 36 gw was lower in the IFA group than in both the LNS group (*P* < 0.001) and the MMN group (*P* = 0.025). Similarly, mean sTfR at 36 gw was lower in the IFA group compared with either the LNS (*P* < 0.001) or MMN group (*P* = 0.046) in models adjusted for baseline sTfR.

**Table 2:** Mean [95% CI] Hb, ZPP, and sTfR at baseline and 36 gestational weeks among pregnant Malawian women, by intervention group

Outcome	Time point	Result by intervention group^d^				Comparison between LNS group and IFA group		Comparison between LNS group and MMN group		Comparison between MMN group and IFA group	
		IFA	MMN	LNS	*P* value^c^	Difference in means^c^	*P* value^c^	Difference in means^c^	*P* value^c^	Difference in means^c^	*P* value^c^
Hb (g L^−1^)	Baseline^a^^,^ e	111.3 [109.8, 112.9]	111.4 [109.9, 112.9]	111.7 [110.2, 113.2]							
	36 gw, adjusted model^b^^,^ e	112.7 [111.2, 114.1]	110.3 [108.8, 111.7]	110.0 [108.5, 111.4]	0.020	−2.7 [−5.2, −0.2]	0.030	−0.3 [−2.7, 2.2]	0.960	−2.4 [−4.9, 0.1]	0.058
	36 gw, after excluding those with CRP >5 g L^−1^ or AGP >1 mg L^−1^ d	113.4 [111.6, 115.2]	111.2 [109.5, 112.9]	111.0 [109.3, 112.8]	0.112	−2.4 [−5.4, 0.6]	0.149	−0.2 [−3.1, 2.8]	0.992	−2.2 [−5.2, 0.7]	0.179
ZPP (μmol mol^−1^ haem)	Baseline^a^^,^ f	46.0 [43.9, 48.2]	46.8 [44.6, 49.2]	46.0 [43.8, 48.4]							
	36 gw, adjusted model^b^	48.9 [46.7, 51.1]	52.0 [49.8, 54.3]	56.5 [54.1, 59.1]	<0.001	9.7 [3.7, 15.7]	<0.001	6.7 [−0.7, 12.7]	0.124	3.0 [−3.0, 9.0]	0.025
sTfR (mg L^−1^)	Baseline^a^^,^ f	4.3 [4.2, 4.5]	4.3 [4.1, 4.4]	4.4 [4.2, 4.6]							
	36 gw, adjusted model^b^	4.8 [4.7, 5.0]	5.1 [5.0, 5.3]	5.3 [5.1, 5.5]	<0.001	0.5 [0.1, 0.9]	<0.001	0.05 [−0.4, 0.5]	0.332	0.5 [0.1, 0.9]	0.046

*Note*. AGP: α‐1‐acid glycoprotein; CRP: C‐reactive protein; Hb: haemoglobin; IFA: iron–folic acid; LNS: lipid‐based nutrient supplement; MMN: multiple micronutrients; sTfR: soluble transferrin receptor; ZPP: zinc protoporphyrin.

a Unadjusted model.

b *P* values for adjusted models shown herein were adjusted only for the baseline value of the outcome variable. Models that included other covariates significantly related to the outcome variable were also examined but did not yield significantly different results than models including only the baseline value of the outcome variable as a covariate.

c Unadjusted *P* values were calculated using ANOVA; adjusted *P* values were calculated using analysis of covariance (ANCOVA). Data for ZPP and sTfR were log‐transformed before analyses were performed. Because of the difficulty in calculating variance of log‐transformed variables, pairwise differences (and 95% CI) for ZPP and sTfR were determined using untransformed data. However, *P* values for the differences were determined using log‐transformed ZPP and sTfR.

d Comparisons between intervention groups among women without inflammation were adjusted for baseline Hb, CRP, and AGP.

e Values reported are mean for unadjusted models and least squares mean for adjusted models.

f Sample sizes: Hb at baseline: IFA *n* = 460, MMN *n* = 463, LNS *n* = 454. Hb at 36 gw: IFA: *n* = 342, MMN: *n* = 353, LNS: *n* = 346. ZPP at baseline: IFA: *n* = 446, MMN: *n* = 445, LNS: *n* = 434. ZPP at 36 gw: IFA: *n* = 330, MMN: *n* = 343, LNS: *n* = 335. sTfR at baseline: IFA: *n* = 456, MMN: *n* = 462, LNS: *n* = 453. sTfR at 36 gw: IFA: *n* = 352, MMN: *n* = 363, LNS: *n* = 352.

Table 3 shows that there were no differences in the prevalence of anaemia, high Hb, high sTfR, or IDA (using a cut‐off of 100 g L^−1^) between groups after adjusting for the baseline status. There were differences between intervention groups in prevalence of iron deficiency when defined by high ZPP but not when defined by high sTfR. Specifically, there was a greater risk of high ZPP among women in the LNS group compared with both the IFA (RR [95% CI]: 1.86 [1.22, 2.83]) and MMN (RR: 1.69 [1.12, 2.56]) groups after adjusting for baseline ZPP.

**Table 3:** Number (percentage) of pregnant Malawian women with abnormal haemoglobin and iron status at baseline and 36 weeks of gestation by intervention group, and pairwise relative risks (RRs) between groups

Outcome	Time point	Number of outcomes/women with outcome data				Comparison between LNS group and IFA group		Comparison between LNS group and MMN group		Comparison between MMN group and IFA group	
		IFA	MMN	LNS	*P* value^d^	Relative risk [95% CI]	*P* value^e^	Relative risk [95% CI]	*P* value^e^	Relative risk [95% CI]	*P* value^e^
Hb <100 g L^−1^	Baseline^a^	97/460 (21.1%)	92/463 (19.9%)	97/454 (21.4%)							
	36 gw^b^	56/342 (16.4%)	76/353 (21.5%)	80/346 (23.1%)	0.087	1.55 [0.96, 2.48]	0.079	0.90 [0.57, 1.40]	0.837	1.39 [0.86, 2.24]	0.242
	36 gw, after excluding those with CRP >5 g L^−1^ or AGP >1 mg L^−1^ c	31/228 (13.6%)	48/246 (19.5%)	52/234 (22.2%)	0.104	1.35 [0.86, 2.13]	0.193	0.88 [0.59, 1.30]	0.516	1.54 [0.99, 2.40]	0.057
Hb >130 g L^−1^	Baseline^a^	55/460 (12.0%)	48/463 (10.4%)	50/454 (11.0%)							
	36 gw^b^	36/342 (10.5%)	26/353 (7.4%)	26/346 (7.5%)	0.329	0.72 [0.38, 1.37]	0.454	0.97 [0.49, 1.92]	0.993	0.70 [0.37, 1.32]	0.385
	36 gw, after excluding those with CRP >5 g L^−1^ or AGP >1 mg L^−1^ c	19/228 (8.3%)	24/246 (9.8%)	20/234 (8.6%)	0.358	1.25 [0.68, 2.29]	0.467	1.15 [0.64, 2.09]	0.639	1.08 [0.58, 2.03]	0.801
ZPP >60 μmol mol^−1^ haem	Baseline^a^	103/446 (23.1%)	114/445 (25.6%)	108/434 (24.9%)							
	36 gw^b^	94/330 (28.5%)	112/343 (32.8%)	136/335 (40.6%)	<0.001	1.86 [1.22, 2.83]	0.002	1.69 [1.12, 2.56]	0.007	1.09 [0.71, 1.68]	0.875
sTfR >6.0 mg L^−1^	Baseline^a^	88/456 (19.3%)	92/462 (19.9%)	90/453 (19.9%)							
	36 gw^b^	103/352 (29.3%)	126/363 (34.7%)	128/352 (36.4%)	0.139	1.41 [0.94, 2.12]	0.119	0.88 [0.59, 1.31]	0.729	1.24 [0.82, 1.87]	0.433
Iron deficiency anaemia^f^	Baseline^a^	49/458 (10.7%)	58/461 (12.6%)	55/451 (12.2%)							
	36 gw^b^ (cut‐off = 100 g L^−1^)	34/339 (10.0%)	51/350 (14.6%)	54/343 (15.7%)	0.079	1.62 [1.05, 2.48]	0.029	1.12 [0.78, 1.61]	0.535	1.44 [0.94, 2.21]	0.096
	36 gw^b^ (cut‐off = 110 g L^−1^)	69/339 (20.5%)	99/350 (28.3%)	103/343 (30.1%)	0.008	1.43 [1.11, 1.85]	0.006	0.92 [0.73, 1.14]	0.434	1.31 [1.02, 1.70]	0.038

*Note*. AGP: α‐1‐acid glycoprotein; CRP: C‐reactive protein; Hb: haemoglobin; IFA: iron–folic acid; LNS: lipid‐based nutrient supplement; MMN: multiple micronutrients; sTfR: soluble transferrin receptor; ZPP: zinc protoporphyrin.

a Unadjusted model.

b *P* values for adjusted models shown herein were adjusted only for the baseline value of the outcome variable. Models that included other covariates significantly related to the outcome variable were also examined but did not yield significantly different results than models including only the baseline value of the outcome variable as a covariate.

c Comparisons between intervention groups among women without inflammation were adjusted for baseline Hb, CRP, and AGP.

d Unadjusted *P* values were calculated using Fisher's exact test; adjusted *P* values were calculated using logistic regression models.

e Adjusted *P* values were calculated using log‐Poisson regression models.

f Hb <100 and either ZPP >60 μmol mol^−1^ haem or sTfR >6.0 mg L^−1^.

When using a cut‐off of 110 g L^−1^ to define anaemia and IDA, there continued to be no differences in the prevalence of anaemia between intervention groups (global *P* = 0.158; data not shown), but the risks of IDA among women in the LNS and MMN groups were significantly higher than in the IFA group (RR [95% CI]: 1.43 [1.11, 1.85] and 1.31 [1.02, 1.70], respectively; Table 3).

There were no significant interactions (*P* < 0.05) between group assignment and potential effect modifiers when the outcome was mean ZPP or sTfR or proportion with elevated ZPP or sTfR at 36 gw. There were significant interactions between group assignment and baseline Hb or sTfR when the outcome was mean Hb or the proportion of women with high Hb at 36 gw (Table 4). However, in stratified analysis, there were no differences in mean Hb or probability of elevated Hb between groups among women who were or were not anaemic at enrolment. There were no differences in mean Hb among those without elevated sTfR at enrolment, but among those with elevated sTfR at enrolment, Hb was greater at 36 gw in the IFA group compared with both the MMN and LNS groups (*P* = 0.020 and *P* = 0.005, respectively). There were no differences in probability of elevated Hb among those with or without high sTfR at enrolment. There was also a significant interaction between group assignment and presence of malaria infection with regard to the proportion of women with low Hb at 36 gw. Specifically, among those with malaria at enrolment, the probability of low Hb at 36 gw was lower in the IFA and MMN groups than in the LNS group (*P* = 0.028 and *P* = 0.014, respectively), but there were no differences among women without malaria at enrolment.

**Table 4:** Significant interactions with effect of intervention on mean Hb and proportions with low or high Hb among Malawian women at 36 gestational weeks, by baseline level of the effect modifiers

Outcome	Effect modifier	Estimated least squares mean [95% CI] or estimated probability of outcome^a^			*P* value for the interaction	*P* value for the difference between groups
		IFA	MMN	LNS		
Hb at 36 gw (g L^−1^)	Anaemic at enrolment				0.026	
	No	115.2 [113.5, 116.9]	112.6 [111.0, 114.3]	113.0 [111.3, 114.6]		0.062
	Yes	104.7 [101.2, 108.3]	102.5 [99.1, 105.9]	100.9 [97.5, 104.3]		0.297

	High sTfR at enrolment				0.003	
	No	113.7 [112.0, 115.4]	112.1 [110.3, 113.8]	112.2 [110.5, 113.9]		0.325
	Yes	112.4 [108.5, 116.3]_a_	105.4 [102.0, 108.8]_b_	103.9 [100.2, 107.6]_b_		0.004
Low Hb (<100 g L^−1^)	Malaria at enrolment				0.023	
	No	0.17 [0.13, 0.22]	0.23 [0.18, 0.28]	0.20 [0.15, 0.25]		0.290
	Yes	0.15 [0.08, 0.25]_b_	0.13 [0.08, 2.22]_b_	0.32 [0.23, 0.43]_a_		0.005
High Hb (>130 g L^−1^)	Anaemic at enrolment				0.024	
	No	0.11 [0.07, 0.15]	0.08 [0.05, 0.11]	0.09 [0.06, 0.13]		0.444
	Yes	0.04 [0.01, 0.13]	0.01 [<0.01, 0.09]	<0.01 [<0.01, >0.99]		0.599
	High sTfR at enrolment				0.019	
	No	0.09 [0.06, 0.13]	0.06 [0.04, 0.10]	0.05 [0.03, 0.09]		0.101
	Yes	0.10 [0.05, 0.21]	0.07 [0.03, 0.15]	0.06 [0.02, 0.14]		0.101

*Note*. The models for Hb at 36 gw were adjusted for log‐AGP at enrolment, the season when enrolled, and site of enrolment. The models for low Hb were adjusted for log‐ZPP at enrolment. The models for high Hb were adjusted for the season when enrolled. AGP: α‐1‐acid glycoprotein; Hb: haemoglobin; IFA: iron–folic acid; LNS: lipid‐based nutrient supplement; MMN: multiple micronutrients; sTfR: soluble transferrin receptor; ZPP: zinc protoporphyrin. Subscripts with nonmatching letters are significantly different (*P* < 0.05).

a Estimated least squares mean (95% confidence interval) Hb at 36 gw, or estimated probability of low or high Hb among those with and without anaemia, elevated sTfR, or malaria. Analyses are based on ANCOVA (SAS PROC GLM, with SLICE option) for continuous outcomes or logistic regression (SAS PROC GLIMMIX, with SLICE option) for binary outcomes.

We also examined differences in mean Hb and proportions of women with low or high Hb among women without inflammation (CRP <5 mg L^−1^ and AGP <1 g L^−1^). Of the 1,026 women from whom Hb, CRP, and AGP data were available at 36 gw, 318 had inflammation and were excluded. We found no differences between intervention groups in mean Hb at 36 gw (*P* = 0.112; Table 2) or prevalence of anaemia (*P* = 0.104; Table 3) after adjusting for baseline Hb, CRP, and AGP. There were no differences between the intervention groups in the proportion of women with elevated Hb (*P* = 0.358) after excluding women with inflammation in adjusted models.

## 4 DISCUSSION

Among pregnant Malawian women enrolled in the iLiNS‐DYAD study, those who were provided with IFA (60 mg of iron per day) from enrolment (≤20 gw) to 36 gw had higher Hb and markers of iron status at 36 gw compared with those provided with LNS or MMN (20 mg of iron per day). Furthermore, the prevalence of anaemia tended to be lower in the IFA group compared with the MMN and LNS groups, which corresponds with greater iron deficiency (high ZPP) in the LNS and MMN groups compared with the IFA group, although there were no apparent differences in high sTfR.

These results are similar to those of the sister iLiNS‐DYAD trial in Ghana that had the same study design and interventions as the study in Malawi (Adu‐Afarwuah et al., 2016). In both sites, Hb and iron status were higher among those provided with IFA, compared with LNS and MMN. However, in Ghana, the prevalence of anaemia (Hb <100 g L^−1^) decreased during pregnancy and was quite low (2–8%) by 36 gw, whereas in Malawi, the prevalence of anaemia was similar at enrolment and 36 gw and was higher at 36 gw (~20% at 36 gw) than in Ghana. At the same time, the prevalence of high Hb increased during pregnancy in Ghana, yet decreased in Malawi. There were a number of differences between the Ghana and Malawi populations that may have contributed to the differences in prevalence of low and high Hb. At enrolment, the Ghanaian women had higher Hb, lower prevalence of anaemia and iron deficiency, lower prevalence of malaria, younger age, higher BMI, higher education level, and higher socio‐economic status, and were more likely to be nulliparous.

One obvious explanation for the higher mean Hb and iron status in the IFA group compared with the LNS and MMN groups at 36 gw is that the dose of iron in the IFA was 3 times greater than in the MMN or LNS. Other studies in a variety of populations have shown no differences in Hb or iron status between women who consumed 30 mg of iron in MMN compared with those who consumed 60 mg of iron together with folic acid (Allen, Peerson, & Maternal Micronutrient Supplementation Study, 2009; Mei et al., 2014; Roberfroid et al., 2011). It is possible that the extra 10 mg of iron (30 mg in the latter studies vs. 20 mg in the LNS and MMN used in the current study) accounts for the difference in results. In Australia, Zhou et al. (2009) found that a 20‐mg daily dose of iron was adequate to prevent iron deficiency during pregnancy, but in Denmark, pregnant women who consumed 20 mg of iron per day had a higher prevalence of iron deficiency and IDA compared with those who consumed 40 mg day^−1^ (Milman et al., 2005), which is in line with our findings. Given the high intake of plant‐based foods in Malawi (Ndekha, 1998), it is possible that 20 mg day^−1^ is not an adequate supplement for this population, as the phytate in certain plant‐based foods inhibits iron absorption.

There was a greater prevalence of IDA in the LNS and MMN groups compared with the IFA group when a cut‐off of 110 g L^−1^ was used instead of 100 g L^−1^. This could signify that a higher cut‐off is better able to detect IDA among women who have moderate IDA. Or it could falsely diagnose healthy women as having IDA. Our original plan was to use 100 g L^−1^ as the cut‐off, as has been suggested by WHO and International Nutritional Anemia Consultative Group for adequate sensitivity and specificity in screening for IDA among pregnant women of African descent (Nestel & INACG Steering Committee, 2002; WHO, 2007; WHO/UNICEF/UNU, 2001). Without analysis of functional outcomes associated with IDA, the best cut‐off to use in this population is not known.

There were no apparent differences between the LNS and MMN groups in mean values of Hb or markers of iron status, but there was a higher prevalence of elevated ZPP (but not sTfR) at 36 gw in the LNS compared with the MMN group. Some substances in the LNS but not present in the MMN may have inhibited iron absorption from the LNS, such as calcium (280 mg per serving) and phytic acid (from peanuts). In nonpregnant, multiparous Chilean women, average (±1 *SD*) absorption of iron from iron sulfate alone was 25.0% (11.9% to 52.2%), compared with 13.2% (7.1% to 24.6%) when consumed with calcium and phytic acid (Jaramillo et al., 2015).

Although there was no apparent main effect of intervention group on prevalence of anaemia at 36 gw, there were interaction effects that were significant. Among women who were iron deficient at enrolment (as indicated by high sTfR), Hb was higher at 36 gw among women in the IFA group compared with both the LNS and MMN groups. This suggests that iron deficient women may benefit more from the higher dose of iron in the IFA supplements. Among those with malaria at enrolment, the probability of anaemia at 36 gw was greater in the LNS group compared with either the IFA or MMN group. Although this may be a spurious finding, it is possible that malaria‐induced inflammation inhibited iron absorption, which was already potentially an issue for the LNS group.

Strengths of the study include random allocation of participants, blinding of group assignment among staff involved in data collection, laboratory analyses, and statistical analyses, and rigorous quality control during sample collection and laboratory analysis. Given the difference in participant characteristics between those who were lost to follow‐up and completed follow‐up, these study findings may not be generalizable to all individuals in the study catchment area. However, there were no differences between intervention groups in the proportion lost to follow‐up that may have altered the interpretation of the effect of intervention on Hb and iron status. Because we excluded women with Hb <50 g L^−1^, the results may also not be generalizable to women with severe anaemia. Another limitation of the study is that we relied on participant reporting of supplement consumption rather than direct observation. We were also limited by the inability to blind study staff and participants from knowing who was in the LNS group. However, field workers did not know the difference between MMN and IFA capsules, and all other study staff, laboratory staff, and statisticians were blinded to group allocation until after all laboratory and statistical analyses were performed. Because of the study design, women did not start taking supplements until almost 17 gw, on average. Finally, although we considered various maternal and environmental factors as potential effect modifiers, we did not have information on other factors, such as preconceptional iron status or genetic variation among this population that may have limited iron absorption.

In summary, in this population of pregnant Malawian women, provision of 60 mg of iron in IFA increased mean Hb and markers of iron status but did not appear to reduce the prevalence of anaemia in comparison with provision of LNS or MMN containing 20 mg of iron. Further research is needed to determine (a) why there was no evidence of a reduced prevalence of anaemia at 36 gw with iron supplementation in this population and (b) if higher Hb or iron status later in pregnancy is beneficial with regard to health outcomes of the mother or infant. With regard to the latter issue, although Hb status among these women at enrolment (≤20 gw) was positively associated with birth outcomes (duration of gestation, birthweight, length‐for‐age *z*‐score, and head circumference), there were no associations between Hb at 36 gw and birth size (Dewey & Oaks, 2017). In similar trials in Ghana and Bangladesh, higher iron status at 36 gw, as indicated by low sTfR, was associated with lower birthweight, length‐for‐age z‐score, and head circumference (Dewey & Oaks, 2017), and evidence from other studies also suggests that elevated Hb and iron status in later pregnancy are associated with adverse birth outcomes (Dewey & Oaks, 2017; Steer, Alam, Wadsworth, & Welch, 1995). Therefore, further evaluation of the optimal dose of supplemental iron during pregnancy is warranted.

## CONFLICTS OF INTEREST

The authors declare that they have no conflicts of interest.

## CONTRIBUTIONS

The authors' responsibilities were as follows: PA, UA, KM, and KGD designed research; JMJ, PA, UA, AG, KM, and MN conducted research; LMB performed laboratory analyses; JMJ analysed data; JMJ wrote the paper, with critical input and comments from all other authors; JMJ and KGD had primary responsibility for final content. All authors read and approved the final manuscript.

## Supporting information

Table S1. Nutrient and energy contents of dietary supplements consumed by women enrolled in the iLiNS Project.
Table S2. Baseline characteristics of pregnant Malawian women included and excluded from statistical analyses of outcomes at 36 gestational weeks.
