# Supplementary material for: Effects of lipid‐based nutrient supplements or multiple micronutrient supplements compared with iron and folic acid supplements during pregnancy on maternal haemoglobin and iron status
Source: Matern Child Nutr. 2018 Jul 26;14(4):e12640. doi: 10.1111/mcn.12640 (PMC6175407; doi:10.1111/mcn.12640)
Supplement: Supplementary file 1 — Table S1. Nutrient and energy contents of dietary supplements consumed by women enrolled in the iLiNS Project. Table S2. Baseline characteristics of pregnant Malawian women included and excluded from statistical analyses of outcomes at 36 gestational weeks. [file MCN-14-e12640-s001.docx]

**Supplemental Table 1.** Nutrient and energy contents of dietary supplements consumed by women enrolled in the iLiNS Project.

| **Nutrient** | IFA | MMN | LNS |
| --- | --- | --- | --- |
| Ration (g/day) | 1 tablet | 1 tablet | 20 g sachet |
| Total energy (kcal) | 0 | 0 | 118 |
| Protein (g) | 0 | 0 | 2.6 |
| Fat (g) | 0 | 0 | 10 |
| Linoleic acid (g) | 0 | 0 | 4.59 |
| α-Linolenic acid (g) | 0 | 0 | 0.59 |
| Vitamin A (μg RE) | 0 | 800 | 800 |
| Vitamin C (mg) | 0 | 100 | 100 |
| Vitamin B1(mg) | 0 | 2.8 | 2.8 |
| Vitamin B2 (mg) | 0 | 2.8 | 2.8 |
| Niacin (mg) | 0 | 36 | 36 |
| Folic acid (μg) | 400 | 400 | 400 |
| Pantothenic acid (mg) | 0 | 7 | 7 |
| Vitamin B6 (mg) | 0 | 3.8 | 3.8 |
| Vitamin B12 (μg) | 0 | 5.2 | 5.2 |
| Vitamin D (µg) | 0 | 10 | 10 |
| Vitamin E (mg) | 0 | 20 | 20 |
| Vitamin K (μg) | 0 | 45 | 45 |
| Iron (mg) | 60 | 20 | 20 |
| Zinc (mg) | 0 | 30 | 30 |
| Cu (mg) | 0 | 4 | 4 |
| Calcium (mg) | 0 | 0 | 280 |
| Phosphorus (mg) | 0 | 0 | 190 |
| Potassium (mg) | 0 | 0 | 200 |
| Magnesium (mg) | 0 | 0 | 65 |
| Selenium (μg) | 0 | 130 | 130 |
| Iodine (μg) | 0 | 250 | 250 |
| Manganese (mg) | 0 | 2.6 | 2.6 |

Supplemental Table 2. Baseline characteristics of pregnant Malawian women included and excluded from statistical analyses of outcomes at 36 gestational weeks.

| Characteristic | Included  (n=1067) | Excluded  (n=312) | p-value^1^ |
| --- | --- | --- | --- |
| Mean (SD) maternal age, years | 25.3 (6.1) | 23.9 (6.1) | <0.001 |
| Mean (SD) maternal education, completed years at school | 4.0 (3.4) | 4.1 (3.7) | 0.853 |
| Mean (SD) proxy for socioeconomic status | -0.04 (1.0) | 0.20 (1.2) | <0.001 |
| Proportion of nulliparous women, (n) | 19.7% (210) | 30.0% (92) | <0.001 |
| Mean (SD) BMI, kg/m² | 22.1 (2.8) | 22.3 (2.8) | 0.189 |
| Proportion of women with a low BMI (< 18.5 kg/m²), (n) | 5.7% (60) | 4.5% (14) | 0.478 |
| Proportion of anemic women (Hb < 100 g/l) (n) | 18.6 % (198) | 28.3% (88) | <0.001 |
| Proportion of women with a positive HIV test, (n) | 12.7 % (134) | 17.4 % (46) | 0.056 |
| Proportion of women with a positive malaria test at enrollment (RDT), (n) | 22.8% (243) | 24.7% (77) | 0.494 |

^1^P-value obtained from T-test (comparison of means) or Fisher’s exact test (comparison of proportions)
